# Prevalence and Correlation of Infectious Agents in Hospitalized Children with Acute Respiratory Tract Infections in Central China

**DOI:** 10.1371/journal.pone.0119170

**Published:** 2015-03-09

**Authors:** Jia Liu, Hongwu Ai, Ying Xiong, Fu Li, Zhou Wen, Weiyong Liu, Tongya Li, Kai Qin, Jianguo Wu, Yingle Liu

**Affiliations:** 1 State Key Laboratory of Virology and College of Life Sciences, Wuhan University, Wuhan, China; 2 Department of Clinical Laboratory, Wuhan Children’s Hospital, Wuhan, China; St. Jude Children's Research Hospital, UNITED STATES

## Abstract

Acute respiratory tract infections (ARTIs) are associated with significant morbidity and mortality worldwide, especially in children under the age of 5 years. Almost 2 million children die from ARTIs each year, and most of them are from developing countries. The prevalence and correlation of pathogens in ARTIs are poorly understood, but are critical for improving case prevention, treatment, and management. In this study, we investigated the prevalence and correlation of infectious agents in children with ARTIs. A total of 39,756 children with one or more symptoms, including fever, cough, sore throat, tonsillitis, pharyngitis, herpangina, pneumonia, and bronchiolitis, were enrolled in the study. All patients were hospitalized in Wuhan Children’s Hospital between October 1, 2010 and September 30, 2012, and were evaluated for infectious agents. Pathogens, including *Mycoplasma pneumoniae*, influenza A virus, influenza B virus, adenoviruses, respiratory syncytial virus, parainfluenza virus, *Legionella pneumophila*, *Chlamydophila pneumoniae*, and *Coxiella burnetii*, were screened simultaneously in patient blood samples using anti-pathogen IgM tests. Regression analysis was used to reveal correlations among the pathogens. Our results showed that one or more pathogens were identified in 10,206 patients, and that *Mycoplasma pneumoniae*, adenoviruses, and influenza B virus were the leading infectious agents. Mixed-infections of pathogens were detected in 2,391 cases, with *Mycoplasma pneumoniae* as the most frequent pathogen. The most common agents in the co-infections were *Mycoplasma pneumoniae* and influenza B virus. Regression analysis revealed a linear correlation between the proportion of mixed infections and the incidence of multi-pathogen infections. The prevalence of infectious agents in children with ARTIs was determined. Equations were established to estimate multiple infections by single-pathogen detection. This revealed a linear correlation for pathogens in children with ARTIs. This study provides useful information for improving case prevention and management.

## Introduction

Lower respiratory tract infections (LRTI) (primarily pneumonia) are one of the leading causes of death worldwide in infants and children, especially in developing country. There are approximately almost 2 million children die from ARTIs each year [1–2]. “Typical” bacteria (e.g., Streptococcus pneumonia) as the principal agent of community-acquired pneumonia (CAP) in children have been widely investigated [3]. Recent studies showed that atypical pathogens are also important cause of LRTI resulting in mild to life threatening illness, which should be obtained more attention. The most common atypical pathogens include *Mycoplasma pneumonia(M*. *pneumophila)*, *Legionella pneumophila* (*L*. *pneumophila*), *Chlamydophila pneumoniae* (*C*. *pneumonia*) and respiratory viruses [2]. These atypical pathogens were listed as common agents of CAP in American Community-acquired Pneumonia Diagnosis and treatment guidelines in Adult (2007) and Chinese Community-acquired Pneumonia Management Guidelines in Children (trial, 2007).

*M*. *pneumoniae*, *C*. *pneumoniae*, and *L*. *pneumophila* cause mild, moderate or severe acute respiratory tract infections in children, responsible for 10% to 30% of CAP in children respectively [4]. *M*. *pneumoniae* is a frequent cause of hospitalization among children as young as 2 years of age and can even necessitate ventilatory assistance. *L*. *pneumophila* can occur across all age groups, but *C*. *pneumoniae* has emerged as an important cause of pneumonia in both adults and children as young as 2 years old. *Coxiella burnetii* (*C*. *burnetii*) is the etiologic agent of Q fever, a known pathogen that causes fever, pneumonia, and intravascular infections [5,6]. Studies of CAP have traditionally focused little on viral causes[2]. Currently, viral infections are also involved with 80% of episodes of CAP in children under 2 years old and over 40% of older children [6–11]. The existing studies have showed that respiratory syncytial virus (RSV) and influenza viruses (Flu) are important pathogens among the hospitalized and outpatient children presenting with ARTI [5, 6].Adenovirus virus (ADV) and parainfluenza viruses (PIV) are also associated with a substantial proportion of ALRI in infants and young children [7–9].

The etiology of LRTI can be established in only 30–50% of cases using conventional methods and unidentified etiology causes inappropriate antibiotic usage, antibiotic resistance, unintended adverse reactions and increased cost. Therefore, rapid, sensitive diagnostic methods and pathogen-directed therapy are important. However, Regarding atypical pathogens are difficult to isolate, conventional culture methods require longer test times and a facility able to perform these tests. In addition, the appropriate sample is critical for the aetiologic diagnosis. Sputum, representing lower-airway secretions, can rarely be obtained from children [2,7]. New rapid detection of multiple viruses and bacteria has been developed lately [8,9]. IgM antibody first appeared in the process of infection, but the last time not long. So it was regarded as diagnostic criteria of early infection. Specific IgM appeared in one week, peak within three weeks after infections, thus more valuable for early diagnosis in children based on Chinese Expert Consensus of Diagnosis and Treatment of *Mycoplasma Pneumoniae* in Children.

With advances in methods aimed to detect pathogens, some pediatric patients with ALRT infections are infected simultaneously by multiple pathogens [10,11]. And it is suggested that co-infections are medically relevant, and effective treatment for severe respiratory tract infections ultimately requires diagnosis of all involved pathogens [12]. Interestingly, recent studies have provided statistical evidence that co-infection is not random, and that co-infection occurs more frequently with certain pathogens than with others. However, Studies designed to identify multiple pathogens simultaneously are limited, and information regarding mixed infections is lacking in China. And preferential interactions among specific pathogens remain uncertain [13].

In this study, we analyzed the data collected a total of 39,756 hospitalized children with ARTIs in Wuhan Children’s Hospital, the largest children’s hospital in Central China. To better understand the epidemiological and etiological characteristics of the infections, all blood specimens were tested simultaneously by immunofluorescence assay for IgMs of nine pathogens. In addition, co-infection with specific pathogens and multiple infections among pathogens were investigated. The prevalence and correlation of pathogens in children with ARTIs were identified and may be useful for the prevention and treatment of ARTIs.

## Materials and Methods

### Study Patients

Written informed consent was obtained from the guardians of the children. The study was conducted according to the principles of the Declaration of Helsinki and was approved by the Institutional Review Board of the College of Life Sciences, Wuhan University, in accordance with its guidelines for the protection of human subjects.

Between October 1, 2010 and September 30, 2012, a total of 39,756 hospitalized children aged 0–15 years with respiratory tract infections at Wuhan Children’s Hospital, the largest children’s hospital in Central China, were enrolled in this study prospectively. The respiratory tract infections were divided into upper and lower respiratory tract infections. The symptoms of upper respiratory tract infections include fever, cough, sore throat, tonsillitis, pharyngitis, and herpangina. Pneumonia and bronchiolitis were considered lower respiratory tract infections. Infected patients with one or more of the symptoms were included in the study. Detailed demographic information on age and gender was documented, and laboratory data were collected from the patients’ medical files.

### Specimens

Blood samples were collected from each child who was given a case definition. Specimens were obtained from children before clinical treatment (within 24 h after hospital admission to avoid inclusion of hospital-acquired infections and antibiotic interference). Blood samples were collected in vacuum blood tubes without the addition of anticoagulants and were further clarified after clotting. Serum was stored at −20°C on ice until analysis. Specimens were stored at −70°C if analysis was not possible within 24 h after collection.

### Pathogen Detection

Specimens were tested simultaneously for *M*. *pneumoniae*, influenza A virus (IAV), influenza B virus (IBV), AdV, RSV, PIV, *L*. *pneumophila*, *C*. *pneumonia* and *C*. *burnetii* using indirect immunofluorescent assay through PNEUMOSLIDE IgM (VIRCELL, Spain): Each slide has 10 wells, each containing one of the above agent antigens and cell control. Serum samples were diluted 1:1 with Phosphate Buffered Saline (PBS) then treated with anti-human IgG sorbent. The sorbent treated diluted serum was incubated 90 min at 37°C with the 10 slide wells. The slide washed twice with PBS. A fluorescent secondary IgM antibody (Anti-human IgM/FITC) was added to the wells and incubated at 37°C for 30 min, then washed twice with PBS. If positive an IgM response (greenish yellow fluorescence) is obtained [9]. And patients in whom any one of the targeted pathogens was detected using the above methods were regarded as positive. Cases in which a single pathogen was detected are referred to as mono-infections; cases of two or more pathogens are referred to as co-infections or multiple infections, respectively.

### Statistical Analysis

General data are presented as a percentage (P), logarithm of the percentage [LN(P)], or mean ± SD. Statistical analyses was performed using Statistics Analysis System (SAS) version 9.0, Statistical Product and Service Solutions (SPSS) version 13.0, and Microsoft Excel 2007. Differences in categorical variables between groups were compared by the χ^2^ test. A single-tailed P-value of <0.05 was considered to be statistically significant.

Regression analysis was used to evaluate the correlation between the proportion of mixed infections and the incidence of the pathogens in co-infections or mixed infections. We initially used the one-sample Kolmogorov–Smirnov to test the distributions of LN[P(co-infections)], LN[P(pathogen 1)]*LN[P(pathogen 2)], and LN[P(pathogen 1)]+LN[P(pathogen 2)]). If the parameter showed normal distribution, we then performed a correlation analysis for the variables. For parameters with P <0.01, we subsequently performed a linear regression test.

If we confirmed a linear relationship between the proportion of mixed infections and the incidence of multi-pathogen infections, we defined the following: K = LN[P(co-infections)] / LN[P(pathogen 1)] + LN[(pathogen 2)]. We divided the data into several groups according to the pathogen, month, and age group, and then compared the k coefficients for each pathogen, month, and age group using ANOVA.

## Results

### Prevalence of Respiratory Agents in Children with ARTIs

**Fig 1:**
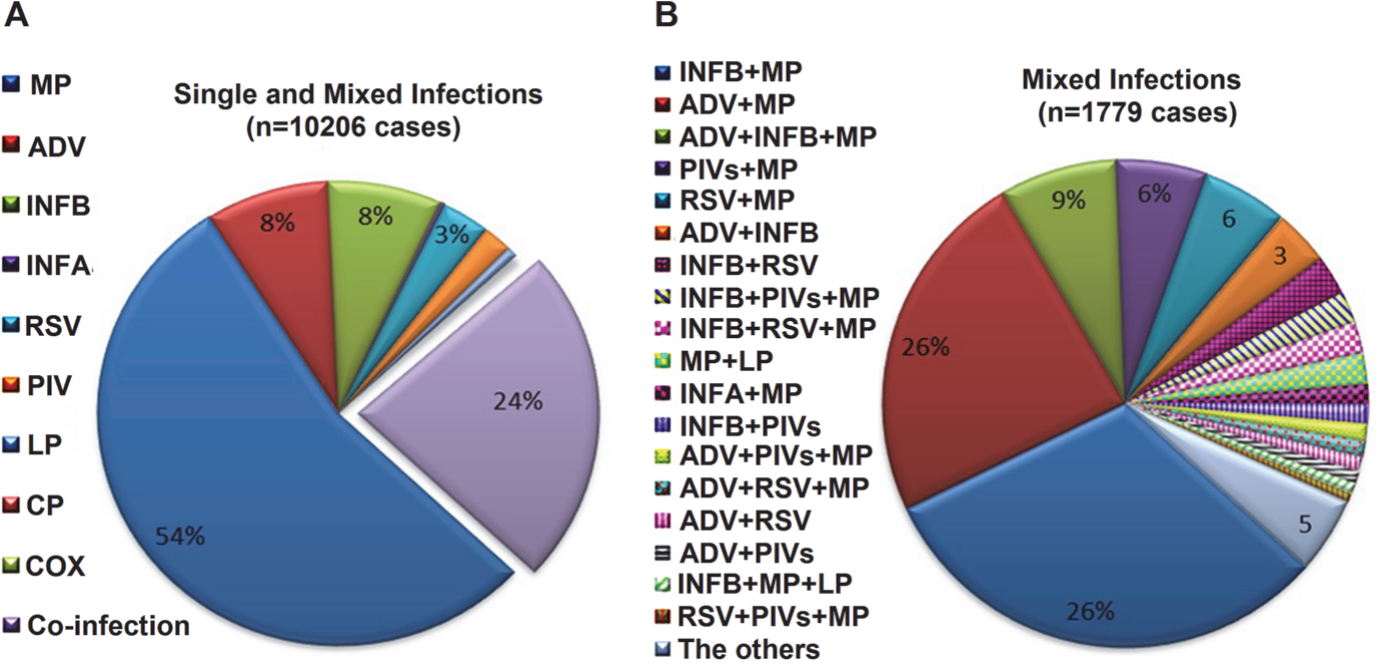
Prevalence of Respiratory Agents in Children with ARTIs. (A) The spectra of single and mixed infections of diverse pathogens in children with ARTIs in Wuhan, China (10,206 cases). (B) The spectrum of mixed infections of diverse pathogens in children with ARTIs in Wuhan (1,779 cases). Note: *Others, including CP+MP (9 cases), INFB+LP (9 cases), AdV+IBV+PIV (9 cases), AdV+IBV+RSV+MP (9 cases), MP+COX (8 cases), AdV+IAV+MP (8 cases), RSV+MP+LP (7 cases), and others (54 cases). Abbreviations: MP, *M*. *pneumonia*; INFB, influenza B virus; INFA, influenza A virus; AdV, adenoviruses; RSV, respiratory syncytial virus; PIV, parainfluenza virus; LP, *L*. *pneumophila*; CP, *C*. *pneumonia*; and COX, *C*. *burnetii*.

Between October 2010 and September 2012, a total of 39,756 children (67.3% males, median age 13 months, range 0–15 years) hospitalized with ARTIs at Wuhan Children’s Hospital were enrolled in this study prospectively (Table 1). Of the 39,756 patients, 25.7% (10,206) were positive for one or more respiratory pathogens (Fig. 1A and Table 1). *M*. *pneumoniae* was the most frequent pathogen (19.1%, n = 7585), followed by AdV (4.8%, n = 1,898), IBV (4.7%, n = 1,851), RSV (2.0%, n = 788), and PIV (1.4%, n = 571). The rates for *L*. *pneumophila* (n = 175), IAV (n = 86), *C*. *pneumonia* (n = 36), and C. burnetii (n = 21) were very low (0.4%, 0.2%, and 0.1%, respectively) (Fig. 1A and Table 1).

**Table 1:** Pathogen Distribution in Children with Acute Respiratory Infection.

Pathogens/Etiology	No. of cases	No. of positive cases	Proportion of total cases	Proportion of positive cases
*M*. *pneumonia*	39756	7585	19.1	74.3
AdV	39756	1898	4.8	18.6
IBVE	39756	1851	4.7	18.1
IAV	39756	86	0.2	0.8
RSV	39756	788	2.0	7.7
PIV	39756	571	1.4	5.6
*L*. *pneumopnia*	39756	175	0.4	1.7
*C*. *pneumonia*	39756	36	0.1	0.4
*C*. *burnetii*	39756	21	0.1	0.2
**Single infections**	**39756**	**7815**	**19.7**	**76.6**
*M*. *pneumoniae*	39756	5515	13.9	54.0
AdV	39756	847	2.1	8.3
IBV	39756	780	2.0	7.6
IAV	39756	34	0.1	0.3
RSV	39756	328	0.8	3.2
PIV	39756	204	0.5	2.0
*L*. *pneumophila*	39756	75	0.2	0.7
*C*. *pneumonia*	39756	22	0.1	0.2
*C*. *burnetii*	39756	10	0.0	0.1
**Multiple infections**	**39756**	**2391**	**6.0**	**23.4**
IBV + *M*. *pneumoniae*	39756	630	1.6	6.2
AdV + *M*. *pneumoniae*	39756	620	1.6	6.1
RSV + *M*. *pneumoniae*	39756	219	0.6	2.1
PIV + *M*. *pneumoniae*	39756	138	0.3	1.4
AdV + IBV	39756	80	0.2	0.8
AdV + PIV	39756	56	0.1	0.5
IBV + RSV	39756	51	0.1	0.5
*M*. *pneumoniae* + *L*. *pneumophila*	39756	48	0.1	0.5
IAV + *M*. *pneumoniae*	39756	34	0.1	0.3
AdV + RSV	39756	33	0.1	0.3
INFB + PIV	39756	26	0.1	0.3
RSV + PIV	39756	18	0.0	0.2
*C*. *pneumonia* + *M*. *pneumoniae*	39756	9	0.0	0.1
IBV + *L*. *pneumophila*	39756	9	0.0	0.1
*M*. *pneumoniae* + *C*. *burnetii*	39756	8	0.0	0.1
AdV + *L*. *pneumophila*	39756	4	0.0	0.0
AdV + IBV + *M*. *pneumoniae*	39756	142	0.4	1.4
IBV + RSV + *M*. *pneumoniae*	39756	40	0.1	0.4
IBV + PIV + *M*. *pneumoniae*	39756	39	0.1	0.4
AdV + PIV + *M*. *pneumonia*	39756	30	0.1	0.3
AdV + RSV + *M*. *pneumonia*	39756	29	0.1	0.3
RSV + PIV + *M*. *pneumoniae*	39756	23	0.1	0.2
IBV + *M*. *pneumoniae* + *L*. *pneumophila*	39756	12	0.0	0.1
AdV + IBV + RSV	39756	10	0.0	0.1
AdV + IBV + PIV	39756	9	0.0	0.1
AdV + IAV + *M*. *pneumoniae*	39756	8	0.0	0.1
RSV + *M*. *pneumoniae* + *L*. *pneumophila*	39756	7	0.0	0.1
AdV + *M*. *pneumoniae* + *L*. *pneumophila*	39756	4	0.0	0.0
AdV + RSV + PIV	39756	4	0.0	0.0
IBV + RSV + PIV	39756	3	0.0	0.0
IAV + PIV + *M*. *pneumonia*	39756	3	0.0	0.0
AdV + PIV + *L*. *pneumophila*	39756	3	0.0	0.0
AdV + IBV + RSV + *M*. *pneumonia*	39756	9	0.0	0.1
IBV + RSV + PIV + *M*. *pneumoniae*	39756	3	0.0	0.0
Others	39756	30	0.0	0.0
**Total**	**39756**	**10206**	**25.7**	**100.0**

Among the positive cases (n = 10,206), 76.6% were mono-infections (n = 7815) and 23.4% were co-infections or multiple infections (n = 2,391). Among the mono-infections, *M*. *pneumoniae* was the leading causative agent (54.0%, n = 5,515). Among the cases of co-infections or multiple infections, 83.5% were co-infections involving two pathogens (n = 1,996), 15.7% were multiple infections involving three pathogens (n = 376), and only 0.8% were multiple infections involving four pathogens (n = 19) (Fig. 1A and Table 1).

*M*. *pneumoniae* was the most frequent pathogen among co-infections and multiple infections (86.8%, 2,070/2,391). *M*. *pneumoniae* plus IBV was the most frequent pathogen combination in dual respiratory infections (26.3%, 630/2,391), followed by *M*. *pneumoniae* plus AdV (17.6%, 420/2,391). *M*. *pneumoniae* plus AdV plus IBV was the most frequent pathogen combination among triple infections (37.8%, 142/376) (Fig. 1B and Table 1). However, the percentage of co-infections was higher for PIV (64.3%, 367/571) compared with IAV (60.5%, 34/86), RSV (58.4%, 460/788), IBV (57.9%, 1071/1851), LP (57.1%, 75/175), AdV (55.4%, 1051/1898), *C*. *burnetii* (52.4%, 11/21), *C*. *pneumonia* (38.9%, 14/36), or *M*. *pneumoniae* (27.3%, 2070/7585) (Fig. 1B and Table 1).

### Monthly Distribution of Respiratory Agents in Children with ARTIs

**Fig 2:**
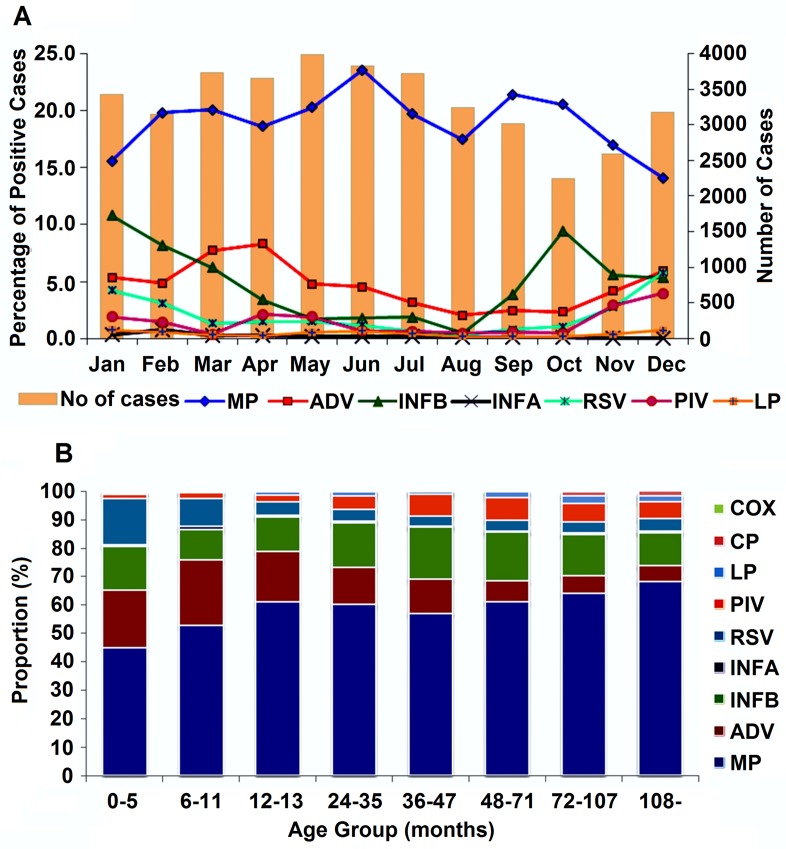
Month and Age Distributions of ARTIs in Children from October 2010 to September 2012. (A) Analysis of the percentage of ARTI cases positive for *M*. *pneumoniae*, adenoviruses, influenza B virus, influenza A virus, respiratory syncytial virus, parainfluenza virus, and *L*. *pneumophila*, and the number of positive ARTI cases in Wuhan, China each month from October 2010 to September 2012. (B) Proportions of different pathogens detected in 39,756 children aged 0 to 15 years (mean age 24.4±29.0 months) with ARTIs in Wuhan from October 2010 to September 2012. Abbreviations: MP, *M*. *pneumonia*; INFB, influenza B virus; INFA, influenza A virus; AdV, adenoviruses; RSV, respiratory syncytial virus; PIV, parainfluenza virus; LP, *L*. *pneumophila*.

Analysis of the monthly distribution of ARTIs revealed that *M*. *pneumoniae*, AdV, IBV, RSV, PIV, and *L*. *pneumophila* were detected throughout the surveillance period (Fig. 2A). *M*. *pneumoniae* was prevalent throughout nearly the entire year, with two small peaks seen in June and September. IBV infection was more prevalent in late autumn and winter, with peaks in January and October. AdV infection was more common in spring and winter, with a peak occurring in April. RSV and PIV occurred more frequently in winter, with a peak in December. *L*. *pneumophila*, *C*. *pneumonia*, and *C*. *burnetii* were detected infrequently and sporadically throughout the year without any obvious seasonal patterns during the study period.

### Sex and Age Distribution of Respiratory Agents among Children with ARTIs

Of the enrolled patients, 26,747 (67.3%) were male and 13,009 (32.7%) were female (Table 2). The percentage who tested positive was significantly higher for females compared with males for both single infections (χ^2^ = 188.7, P<0.001) and co-infections (χ^2^ = 81.4, P<0.001).

**Table 2:** Sex and Age Distributions of Children with Acute Respiratory Infection in Wuhan, China from 2010–2012.

Study group	No. of cases	No. of positive cases (%^#^)										
		*M*. *pneumoniae*	AdV	IBV	IAV	RSV	PIV	*L*. *pneumophila*	*C*. *pneumonia*	*C*. *burnetii*	Single infections	Mixed infections
**Gender**												
Male	26747	4453(16.6)	1220(4.6)	1124(4.2)	52(0.2)	507(1.9)	309(1.2)	95(0.4)	24(0.1)	15(0.1)	4747(17.7)	1408(5.3)
Female	13009	3132(24.1)	678(5.2)	727(5.6)	34(0.3)	281(2.2)	262(2)	80(0.6)	12(0.1)	6(0)	3068(23.6)	983(7.6)
**Age (months)**												
Median	13(5,33)	27(13,49)	16(9,31)	30(13,48)	16(9,35)	12(5,34)	39(24,61)	38(19,71)	84(69,108)	14(8,22)	24(12,47)	27(12,46)
Mean*	24.4±29.0	36.3±31.8	23.6±23.4	35.7±29.6	28.9±30.8	24.3±28.8	45.5±30.1	46.1±32.6	81.9±33.7	21.9±22.8	34.2±31.7	34.2±29.3
Range	0–180	0–180	0–156	0–180	1–156	0–180	0–180	0–156	3–144	4–84	0–180	0–180
0–5	10061	555(5.5)	253(2.5)	188(1.9)	9(0.1)	200(2)	20(0.2)	8(0.1)	1(0)	4(0)	796(7.9)	203(2)
6–11	8464	969(11.4)	425(5)	196(2.3)	19(0.2)	179(2.1)	39(0.5)	5(0.1)	1(0)	5(0.1)	1139(13.5)	324(3.8)
12–23	8086	1915(23.7)	556(6.9)	372(4.6)	24(0.3)	144(1.8)	73(0.9)	42(0.5)	0(0)	7(0.1)	1929(23.9)	555(6.9)
24–35	3832	1222(31.9)	264(6.9)	317(8.3)	13(0.3)	84(2.2)	96(2.5)	30(0.8)	2(0.1)	1(0)	1159(30.2)	396(10.3)
36–47	3047	948(31.1)	199(6.5)	305(10)	6(0.2)	58(1.9)	125(4.1)	18(0.6)	2(0.1)	1(0)	882(28.9)	356(11.7)
48–71	2810	876(31.2)	103(3.7)	247(8.8)	5(0.2)	55(2)	113(4)	29(1)	3(0.1)	1(0)	847(30.1)	272(9.7)
72–107	2122	702(33.1)	66(3.1)	158(7.4)	7(0.3)	42(2)	70(3.3)	31(1.5)	17(0.8)	2(0.1)	667(31.4)	198(9.3)
108-	1334	398(29.8)	32(2.4)	68(5.1)	3(0.2)	26(1.9)	35(2.6)	12(0.9)	10(0.7)	0(0)	396(29.7)	87(6.5)
**Total**	**39756**	**7585(19.1)**	**1898(4.8)**	**1851(4.7)**	**86(0.2)**	**788(2)**	**571(1.4)**	**175(0.4)**	**36(0.1)**	**21(0.1)**	**7815(19.7)**	**2391(6)**

*Mean ± standard deviation (SD)

^#^ The proportion of each gender/age group

The age of the patients ranged from 0 to 15 years, with a mean of 24.4±29.0 months (Fig. 2B). The median age of children with RSV infections was lower than of those with other infections. The most frequent age range of children with ARTIs was 0–5 months, accounting for 25.3% of all enrolled cases; the least frequent ages were those >9 years (3.4%). The rate of respiratory infections was much higher in children older than 1 year compared with younger children [χ^2^ = 2786.9, P<0.001]. *M*. *pneumoniae* occurred frequently in all age groups but was more frequent in children older than 1 year. RSV and AdV infections were much more frequent than other viruses in children younger than 1 year and decreased with age; 66.4% of RSV infections and 65.0% of AdV infections occurred in children younger than 2 years old. IBV was more frequent in children older than 1 year. *C*. *pneumonia* and *C*. *burnetii* were infrequent among all age groups.

### Linear Relationship between the Proportion of Mixed Infections and the Incidence of the Pathogens in Multiple Infections

**Fig 3:**
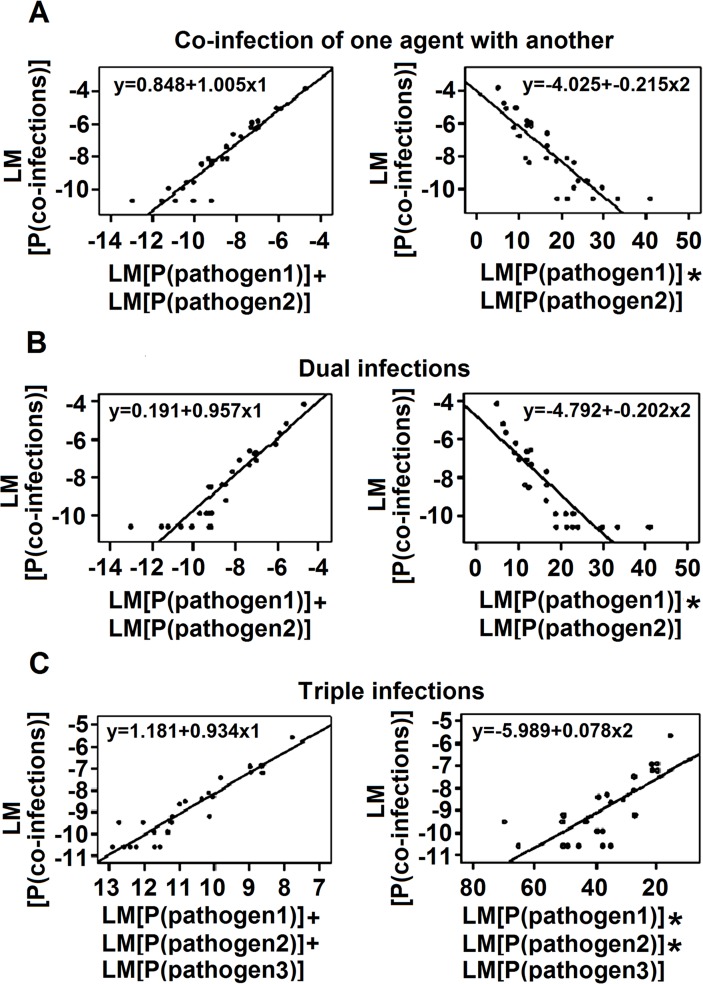
The Linear Relationship between the Proportion of Mixed Infections and the Incidence of Each Pathogen Involved in the Infection. (A) Mixed infections, including triple and quadruple pathogen infections. (B) Co-infections involving two pathogens. (C) Mixed infections involving three pathogens. Notes: Y{LN[P(co-infections)]} denotes the logarithm of the proportion of co-infections for pathogens 1 and 2 in all cases (39,756); X{LN[P(pathogen 1)]} denotes the logarithm of the proportion of pathogen 1 (including single and multiple infections) in all cases (39,756) and LN[P(pathogen 2)] that for pathogen 2; X_1_ denotes {LN[P(pathogen 1)]+LN[P(pathogen 2)]}; and X_2_ denotes {LN[P(pathogen 1)]*LN [P(pathogen 2)]}.

**Fig 4:**
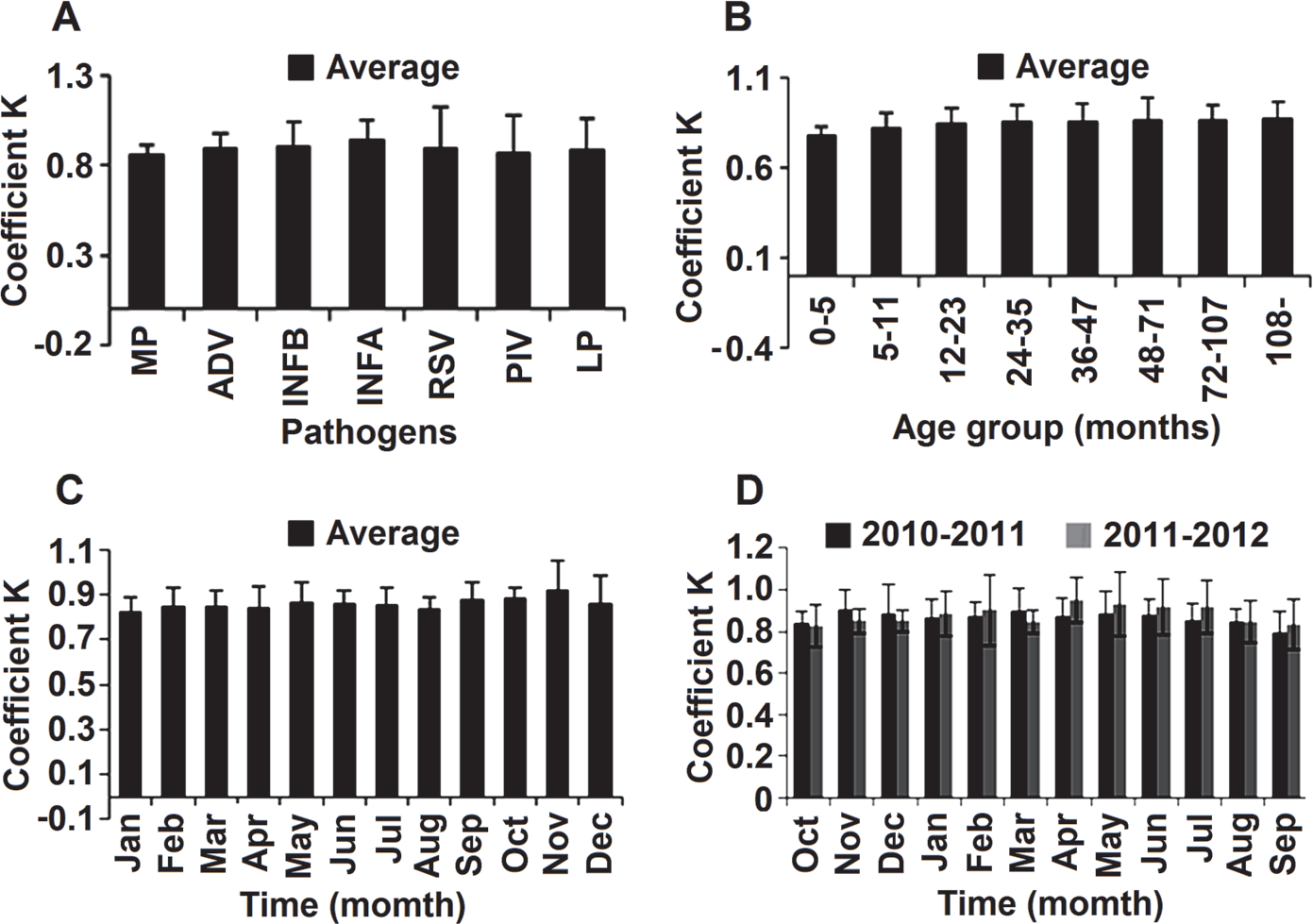
The Average K Coefficient(s) for the Linear Relationship. (A) The average k coefficient for each pathogen. The k coefficients for different pathogens slightly differed from each other, but not significantly (p = 0.971). (B) Variations in the k coefficient by age group. The k coefficients for different age groups slightly differed from each other, ranging from 0.79–0.87 (P = 0.053). (C) The average k coefficient according to seasonal group. The k coefficients for different seasons were very similar (P = 0.285). The maximum D-value for k coefficients for the different seasons was 0.09. (D) Variation in k coefficient by month during the study period. The k coefficients for different months were similar, with no statistically significant differences (P = 0.499). Note k coefficients = y/x = LN[P(co-infections)] / (LN[P(pathogen 1)] + LN[(pathogen 2)]).

Regression analysis showed a linear correlation between the percentage of co-infections and the proportion of each agent detected in the co-infection. We analyzed the correlations of LN[P(co-infections)] (y) with {LN [P(pathogen 1)] + LN [P(pathogen 2)]} (x_1_) and {LN[P(pathogen 1)]*LN [P(pathogen 2)]} (x_2_). When we grouped co-infection by one pathogen with that of another, the functions were y = 0.848+1.005x_1_ (R^2^ = 0.901) and y = −4.025+ −0.215x_2_ (R^2^ = 0.738) (Fig. 3A). The linear correlation remained in dual infections, with the functions y = −0.191+0.957x_1_ (R^2^ = 0.886) and y = −4.792–0.205x_2_ (R^2^ = 0.731) (Fig. 3B). For triple infections, the equations were y = 1.181+0.934x_1_ (R^2^ = 0.876) and y = −5.989+0.078x_2_ (R^2^ = 0.571) (Fig. 3C). Since quadruple infections were only confirmed in 19 patients, the above regression relationship could not be confirmed in cases infected with four pathogens simultaneously. We compared the R^2^ values for the two types of equation and found that the representativeness of the functions for y and x_1_ was higher than that for y and x_2._ We therefore selected x_1_ as the independent variable for further analysis. ANOVA demonstrated no significant difference among various k coefficients for different agents, seasons, and age groups (Fig. 4).

Further regression analysis showed a linear relationship between the proportion of mixed infections and the incidence of the pathogen lower in proportion than the other pathogen involved in the co-infection (Table 3). We could estimate multiple infections by single-pathogen detection using the equations.

**Table 3:** The Linear Relationship between the Proportion of Mixed Infections and the Incidence of the Individual Pathogens Involved in Co-infections.

Multiple infections		Functions	R^2^	P value
Pathogen 1	Pathogen 2			
*M*. *pneumoniae*	AdV	y = −0.768+1.058x	0.734	0.000
*M*. *pneumoniae*	IBV	y = −1.396+0.777x	0.944	0.000
*M*. *pneumoniae*	RSV	y = −0.644+1.058x	0.950	0.000
*M*. *pneumoniae*	PIV	y = −1.515+0.847x	0.766	0.000
*M*. *pneumoniae*	*L*. *pneumophila*	y = −1.396+0.777x	0.944	0.000
AdV	PIV	y = −0.226+1.399x	0.883	0.000
AdV	RSV	y = −1.226+1.300x	0.732	0.002
RSV	PIV	y = −1.768+1.185x	0.751	0.003

Notes: y denotes the logarithm of the proportion of co-infections for two given pathogens in all cases (39756); x indicates the logarithm of the percentage of the lower-proportion pathogen involved (single and multiple infections) in co-infections; P values were determined by ANOVA.

## Discussion

In this study, we found that 25.7% of the inpatient children aged <16 years with ARTIs were infected by at least one respiratory pathogen. *M*. *pneumoniae*, IBV, and AdV were the predominant agents. Multi-pathogen infections were also detected, with *M*. *pneumoniae* as the most frequently involved agent. The most common pathogens in co-infections were *M*. *pneumoniae* and IBV. Regression analysis showed a linear correlation between the percentage of co-infections and the proportion of each agent in the co-infection. We showed that *M*. *pneumoniae* is a major cause of respiratory infections in school-age children and young adults. Recent reports from several European countries have indicated an increase in the detection of *M*. *pneumoniae* infection over the past few years, notably in children aged 4–15 years [14–17]. *M*. *pneumoniae* infection accounted for 32.3% cases of respiratory infection in Wuhan, China [18]. In most studies, RSV was the leading cause of respiratory tract infections, especially in hospitalized infants less than 6 months of age [19–21]. In agreement with these studies, RSV was detected in only 2% of cases in this study.

Influenza viral infections were also common, in that 18.1% of cases were caused by these viruses, with IBV as the major agent. Generally speaking, simultaneous IAV and IBV infections were prevalent during the study period [22,23]. However, IAV has been reported to be the more frequent agent [24], and IBV epidemics occur in approximately 3- to 4-year cycles [25]. AdV infections were also found in respiratory tract infections. Several studies in China revealed that the proportion of AdV infections is increasing [26–28]. In this study, AdV was detected in 4.8% of ARTIs in children less than 16 years of age. This finding suggests that AdV is an important pathogen of ARTIs in inpatients in Wuhan that should receive more attention.

It has been suggested that most *M*. *pneumoniae* epidemics occur in either summer or autumn, with no obvious explanation for this seasonal variation [29–31]. In this study, *M*. *pneumoniae* was prevalent throughout almost the entire year, with peaks occurring in June and September. An epidemiologic study found that influenza viral infection occurred throughout the year with no seasonal predominance [32]. This study showed that influenza viral infection was more prevalent in late autumn and winter.

Our data appear to be in agreement with reports that the rate of ARTI is rising in older children [18,20,24]. This is probably related to the increased risk of infection in school-age children due to encounters with contagious individuals [24]. It may also be due to waning levels of passively transferred maternal antibodies [33]. In the majority of studies, *M*. *pneumoniae* was found in all age groups, with a higher prevalence in children aged 5–14 years [15–17,34]. We detected *M*. *pneumoniae* in all age groups, with greater frequency in children older than 2 years. In our study, the median age of children with RSV infections was lower than that of children with other infections, and AdV infections decreased with age.

The proportions of mixed infections reported in children with ARTIs vary greatly, ranging from 2 to 50%. This is due to diverse pathogens, test methods, and study designs. However, the most frequent mixed infections involved two different pathogens [18,20–22,35,36]. In this study, we found that the prevalence of co-infections was 6.0%. It has been suggested that influenza is the virus most frequently involved in co-infections in Wuhan [18], while AdV is most frequently involved in co-infections in Taiwan [22]. This difference may be due to differences in pathogen epidemiology, study populations, and/or the time the study was conducted.

Recent studies have provided statistical evidence that co-infection is not random, and that co-infection with certain pathogens occurs more frequently than that with other pathogens [18,37]. The mechanism of multi-pathogen infections is still not clear. Some studies suggest that extensive damage to the epithelium of the respiratory tract in some viral ARTIs might promote superinfection by another virus [38]. Whether infection by one agent facilitates infection of the same pathogen or other pathogens in cells is still uncertain. We revealed a linear relationship between the proportion of mixed infections and the incidence of multi-pathogens infections. The k coefficients showed no significant differences according to pathogen, month, or age group. We may use the k coefficient as a constant in further studies. Our study supports the hypothesis that the proportion of the specific pathogen, rather than the pathogen itself, is relevant for co-infections.

Some reports showed that the clinical spectra for co-infections were more severe than those for single infections [20,39–41]. It was proposed that effective treatment for severe ARTIs will ultimately require identification of all involved pathogens [37]. Because routine identification of the causative agent in patients with respiratory infections is not cost-effective, information regarding mixed pathogen infections is lacking [42,43,44]. If further studies confirm that the linear relationship is also relevant to other pathogens or diseases in various regions, we will be able to estimate multiple infections by single-pathogen detection. However, the formula will work best for large samples. Nonetheless, it will help clinicians and researchers involved in the treatment, prevention, and control of ARTI.

This study is representative of children < 16 years of age with ARTIs in Wuhan during the period evaluated, since we continuously monitor ARTI cases in the largest children’s hospital of Central China using a sensitive, specific immunofluorescence assay. The large number of specimens provided an adequate database, which allowed us to draw meaningful conclusions regarding the frequencies and seasonal distributions of the agents. Regrettably, because the amount of data was so immense, it was too difficult to collect clinical information.

In conclusion, our study clarified the spectra of causative agents of ARTIs in inpatient children in Wuhan. *M*. *pneumoniae* remains the most common causative pathogen, followed by influenza viruses and AdV. Multiple viral infections were frequent in children with ARTIs. Regression analysis showed a linear relationship between the proportion of mixed infections and the incidence of the pathogens involved in co-infections. Continuous monitoring of agents frequently related to ARTIs should be encouraged in clinical facilities to improve case diagnosis, treatment, and management.
